# 405 nm light microbicidal efficacy on *Treponema pallidum* spiked in ex vivo human platelets

**DOI:** 10.1038/s41598-025-03230-1

**Published:** 2025-06-06

**Authors:** Oksana Yakovleva, Teresa Pilant, Pravin Kaldhone, Joseph Jackson, David Rotstein, Caitlin Stewart, John Anderson, Scott MacGregor, Michelle Maclean, Luisa Gregori, Chintamani Atreya

**Affiliations:** 1 Division of Emerging Transfusion-Transmitted Diseases, Office of Blood Research and Review, Center for Biologics Evaluation and Research, FDA, 10903 New Hampshire Avenue, Silver Spring, MD 20993 USA; 2 Division of Blood Components and Devices, Office of Blood Research and Review, Center for Biologics Evaluation and Research, FDA, Silver Spring, MD 20993 USA; 3 Division of Food Compliance, Office of Surveillance and Compliance, Center for Veterinary Medicine, FDA, Rockville, MD 20855 USA; 4 The Robertson Trust Laboratory for Electronic Sterilization Technologies, Department of Electronic and Electrical Engineering, University of Strathclyde, Glasgow, UK; 5 Department of Biomedical Engineering, University of Strathclyde, Glasgow, UK; 6 Present Address: Congressional Research Service, Library of Congress, Washington, DC 20540 USA

**Keywords:** *Treponema pallidum*, Syphilis, Violet-blue light, Bacterial inactivation, Rabbit, Diseases, Infectious diseases, Bacterial infection

## Abstract

Pathogen reduction technologies using chemicals and or UV light have been demonstrated to improve the safety of ex vivo platelets from infectious diseases. However, UV light exposure also may affect the treated products, depending on wavelength and exposure. Alternatively, visible spectra 405 nm violet-blue light has broad-spectrum microbicidal activity. Here we tested the effect of 405 nm light on *Treponema pallidum*, the bacterium that causes syphilis. We contaminated platelets stored in plasma with two treponemal concentrations (low and high titers) and treated an aliquot with 270 J/cm^2^ dose (irradiance = 15 mW/cm^2^) of 405 nm light while another aliquot remained untreated. Next, we inoculated intradermally an aliquot of both samples into rabbits. Rabbits inoculated with untreated samples developed syphilis while animals inoculated with light-treated samples did not. Thus, inactivation was demonstrated to the limit of detection of the bioassay. We estimated > 2 log_10_ and > 4 log_10_ reduction in the low and high dose studies, respectively. These results provide proof-of-concept that 405 nm light is effective in reducing syphilis risk in ex vivo platelets.

**Supplementary Information:**

The online version contains supplementary material available at 10.1038/s41598-025-03230-1.

## Introduction

The spirochete bacterium *Treponema pallidum* subsp. *pallidum* is the causative agent of syphilis, a disease known under many names since the Middle Ages^1,2^. Syphilis is primarily transmitted by sexual contact; it is highly invasive and presents with multifaceted manifestations during chronic stages^3,4^. Syphilis is easily treatable with antibiotics such as penicillin although its diagnosis and management can be challenging due to the difficulty in interpreting serological diagnostic test results and evaluation of the response to therapy. In the U.S., cases of syphilis have increased in the past 10 years among females, with a concomitant increase in cases of congenital syphilis, and in males, primarily in men who have sex with men^5^. This resurgence of syphilis in high-risk populations is alarming not only in itself but also because of its potential spread to low-risk populations like blood donors. This scenario has been of particular concern to the blood transfusion community since syphilis can be transmitted by blood transfusion^6^. New studies in the U.S. and elsewhere have investigated how the increase of syphilis cases has impacted syphilis risk in current blood donations. The results showed a correlation between the country’s syphilis epidemic and the rise of syphilis positivity in blood donors^7–9^. However, this increase has not resulted in new cases of transfusion-transmission syphilis, and this is due to the effective risk mitigation measures such as, donor screening questionnaire and universal syphilis testing of donated blood that have been in place for more than 70 years^10^. In addition, the conventional practice of refrigerated storage of whole blood provides some additional level of reduction of *Treponema pallidum*, although not as effective and rapid as previously reported^11,12^. Testing all whole blood donations for multiple pathogens, including *Treponema pallidum*, has protected the blood supply, and maintained the safety of blood transfusions. However, this strategy has also resulted in an ever-expanding list of emerging or reemerging pathogens to be tested and in some cases mandated tests are based on regional or temporal risks. Implementation of these complex testing scenarios has increased costs and challenges to the blood industry^13^.

Platelets are the second most transfused blood component as their therapeutic use has grown over the years^14,15^. Platelet transfusion safety is of particular concern because this component is stored at room temperature which supports growth of bacterial contamination. A meta-analysis using published data from studies encompassing multiple countries and preparation methods of platelets estimated an overall contamination rate of 1:2000 units as assessed by primary culture^16^. These results aligned with CDC’s active and passive surveillance data^17^. To mitigate transmission risks of bacterial infections, each platelet unit is assayed using bacterial culture or rapid tests^18,19^. Bacterial culture methods test an aliquot of the platelet unit using optimized conditions for bacterial growth^20^. Those tests are highly sensitive and their main limitations are that detection of low bacterial contamination could require up to 3 days of incubation and certain bacteria do not grow to detectable levels^21^. As an alternative or complementary to detection, bacteria contaminating platelets and plasma can be inactivated or reduced using pathogen reduction technologies (PRTs)^22^. Among available PRTs is Cerus INTERCEPT Blood System that utilizes amotosalen (S-59) and long-wavelength ultraviolet (UV-A) light to cross-link DNA and RNA in a variety of bacteria, viruses, and parasites^23–25^. Another system called Mirasol Pathogen Reduction Technology System from Terumo uses riboflavin and UV-A/B light to block pathogens’ replication^26,27^. UV-C light alone used in THERAFLEX, Macopharma to successfully inactivate multiple pathogens^28^. These technologies were demonstrated effective against a wide range of blood-borne pathogens in ex vivo plasma and platelets, but post-treatment recovery and quality of platelets is technology-dependent, in some studies suboptimal outcome and recovery were reported^29^. To overcome the challenge that hemoglobin absorbs UV-light, alternative PRT compounds have been developed, for example, amustalin (S-303) has shown some promising results with RBCs in two phase III clinical trials^30^. Another viable option for light-based pathogen inactivation is violet-blue 405 nm visible light^31^. Small scale studies demonstrated that violet-blue light treatment of ex vivo platelets and plasma had mild to no effect on platelets function and recovery^32,33^ and on plasma proteins^34^. Importantly, this treatment does not require added photosensitive agents. The established mechanism of inactivation is based on the generation of reactive oxygen species (ROS) within the cellular microbe induced by the photoexcitation of endogenous porphyrin molecules^35,36^. In the case of viruses, it is speculated that flavins and flavin derivatives in the surrounding media contribute to the ROS induction^36^. Induction of ROS in turn induces formation of nonspecific oxidative damage and microbial death^33,34^. This microbicidal method has been tested in the laboratory and proven effective against bacteria, viruses and parasites suspended in either plasma or platelets under experimental conditions^35,37–40^. We report here 405 nm light inactivation of *Treponema pallidum* spiked in human platelets resuspended in plasma using rabbit bioassays to assay treponemal infectivity.

## Results

### 405 nm light inactivation of *Treponema pallidum*

To assess whether 405 nm light inactivated *Treponema pallidum* bacteria spiked in ex vivo human platelets, we conducted three independent studies using platelets from three donors. In Studies 1 and 2, we spiked platelets with final 5 × 10^5^ treponemes(treps)/ml (low titer) and in Study 3, the final spike was 5 × 10^7^ treps/ml (high titer). In each study, we assayed three samples: the initial time point (T0) obtained before starting inactivation, after 5 h (270 J/cm^2^) exposure to 405 nm light with shaking (T5-Inactivated), and after 5 h shaking but no light exposure (T5-Control). The light dose was selected based previous studies demonstrating its effectiveness against different blood pathogens spiked in plasma and in platelets^35,37–40^. We measured treponemal infectivity using intradermal inoculations in rabbits. Each sample was serially diluted, and each dilution was inoculated at two adjacent sites (left and right) on the shaved back of four rabbits. At the end of the study, we scored each site as positive or negative for syphilis as shown in Supplementary Tables S1 and S2. These tables also report the estimated number of treponemes inoculated per site based on darkfield count of the treponemal stock and a schematic of the injected sites’ location in a rabbit. The results for Studies 1 and 2 are in Table 1. All sites inoculated with 10^–1^ dilution of T0, and T5-Control samples developed lesions consistent with syphilis infection (positive) and the breakpoints were between 10^–2^ and 10^–3^ dilutions. Higher dilutions of the inoculum resulted in no infections (negative). T5-Control samples (Studies 1 and 2) had very similar titers at 10^3.4^ ID_50_/ml and 10^3.5^ ID_50_/ml while we observed a small difference in the titers of T0 samples (10^3.3^ ID_50_/ml versus 10^3.6^ ID_50_/ml). In contrast, all sites inoculated with samples exposed to 405 nm light were syphilis negative (T5-Inactivated). To enhance the sensitivity of the assay, we increased the number of sites injected with the highest concentration of inactivated sample (T5-Inactivated) (10^–1^) from eight to 16 in Study 1 and 24 in Study 2. Assuming the worst case, we estimated the titers to be < 10^1.5^ ID_50_/ml for T5-Inactivated samples in both studies. This means that the method reduced infectivity to below the limit of detection of the bioassay or > 2 log_10_ (> 99%).

**Table 1:** Summary results of Studies 1 and 2 with low titer of treponemal spikes. Each dilution was injected at two sites in four rabbits for a total of 8 sites per dilution, except where otherwise noted. Log_10_ dilutions refer to treponemal stock as dilution 0.

log_10_ dilution	Study 1			Study 2		
	T0	T5 Inactivated	T5 Control	T0	T5 Inactivated	T5 Control
− 1	8/8*	0/16	6/6	8/8	0/24	8/8
− 2	6/8	0/8	5/6	7/8	0/8	8/8
− 3	0/8	0/8	0/6	2/8	0/8	0/8
− 4	0/8	–	0/6	0/8	–	0/8
− 5	0/8	–	0/6	0/8	–	0/4
Heat-Inact**	–	0/8	–	–	–	0/4
Titer ID_50_/ml	10^3.3^	< 10^1.5^	10^3.4^	10^3.6^	< 10^1.5^	10^3.5^

*Positive sites/total sites inoculated.

**Heat-inactivated treponemes diluted in human platelets (10^–1^) inoculated at duplicate sites in four rabbits (Study 1) and in two rabbits (Study 2).

We repeated the study with 100-fold higher final treponemal concentration of 5 × 10^7^ treps/ml (Study 3). Table 2 and Supplementary Table S3 report the results for Study 3. The calculated titers of samples T0 and T5-Control were 10^6.2^ ID_50_/ml and 10^5.5^ ID_50_/ml, respectively. A relatively small drop in titer of T5-Control compared to the initial titer was also observed in Study 2 and in similar studies with *Leishmania donovani* parasite^35^. Rabbits inoculated with T5-Inactivated sample developed no skin lesions and were serologically negative with treponemal and nontreponemal tests. As above, assuming the worst case, we calculated the titer to be < 10^1.5^ ID_50_/ml and estimated infectivity reduction to below the limit of detection of animal assay or > 4 log_10_ (> 99.99%).

**Table 2:** Summary results of Study 3 with high titer of treponemal spike. Each dilution was injected at two sites in four rabbits for a total of 8 sites per dilution except where otherwise noted. Log_10_ dilutions refer to treponemal stock solution as dilution 0.

log_10_ dilution	Study 3		
	T0	T5 Inactivated	T5 Control
− 1	–	0/16	–
− 2	–	0/8	–
− 3	8/8*	0/8	8/8
− 4	8/8	0/8	6/8
− 5	5/8	–	2/8
− 6	0/8	–-	0/8
− 7	0/8	–	0/4
Platelets**	–	–	0/4
Titer ID_50_/ml	10^6.2^	< 10^1.5^	10^5.5^

*Positive sites/total sites inoculated.

**Control with human platelets alone inoculated in duplicate sites in two rabbits for a total of four sites.

### Syphilis serology

In addition to scoring skin lesions, we tested rabbit sera during the study for the presence of treponemal and nontreponemal antibodies. All rabbits inoculated with untreated samples (T0 and T5-Control) had syphilis-positive serology results usually three weeks post inoculation while all rabbits inoculated with inactivated materials remained serologically negative two months post injection. To further investigate those results, we tested sera from nonreactive rabbits at 1:2 dilution, instead of 1:40 the recommended dilution, and confirmed their negative status. In contrast, sera from serologically reactive rabbits were positive up to dilution 1:2048. These results demonstrated high sensitivity of the serological test with rabbit sera and confirmed lack of seroconversion of rabbits injected with treated samples.

### Investigation of inoculated sites in rabbit G130

In Study 2, three of the 24 sites inoculated with 10^–1^ dilution of T5-Inactivated sample showed indurations that persisted to the end of the study but appeared different from the syphilitic lesions. All three sites were in rabbit G130 and are marked in red in Supplementary Table S2. The initial assessment of these three sites was inconclusive. Although rabbit G130 never seroconverted, we were uncertain about the final assignment of those three sites and decided to investigate them further. To this goal, we biopsied all inoculated sites in rabbit G130 and as a control we also examined corresponding tissues from rabbit G135, a syphilis infected animal. We processed the tissues from the left sites for histology and the tissues from the right sites for detection of treponemal DNA. We also conducted similar analyses with popliteal lymph nodes known to harbor treponemes in infected animals.

### Histopathological analysis of skin tissues

Hematoxylin and eosin staining of skin from sites inoculated with T5-Control dilutions 10^–1^ and 10^–2^ in rabbit G135 (positive control) showed skin damage exhibiting similar patterns of host response. Supplementary Fig. S1 A-D shows representative staining of tissue at site dilution 10^–1^. Histopathologic finding included full thickness epithelial necrosis, lymphoplasmacytic to granulomatous dermatitis, edema, and vascular endothelial hypertrophy (Supplementary Fig. S1 A-C) and uncommonly lymphoplamacytic to granulomatous myositis (Supplementary Fig. S1 D). We also observed mild follicular atrophy in tissues from dilutions 10^–3^, 10^–4^ and 10^–5^ with dermal edema and vascular endothelial hypertrophy like those reported in Supplementary Fig. S1C, but those sites showed no or mild inflammation. To rule out a hypersensitivity response in skin samples with edema and vascular endothelial cell hypertrophy as sole findings, we stained sections of skin with Giemsa to identify mast cells. No mast cells were observed. We also stained selected tissues with Warthin-Starry, a silver stain known to detect treponemes. Few treponemes were detected in the necrotic epithelium at the infected sites of rabbit G135 (Supplementary Fig. S4). Histological evaluation of sites in rabbit G130 inoculated with T5-Inactivated dilution 10^–3^ displayed epithelium with no evident necrosis or tissue damage (representative example in Supplementary Fig. S2). Supplementary Fig. S3 shows staining of tissue from the inconclusive site (rabbit G130, dilution 10^–1^) indicating mild epithelial hyperplasia and mild to moderate dermal edema and occasional hair follicle rupture with inflammation (furunculosis). Those observations were not consistent with syphilitic lesions and could represent an unrelated hypersensitivity response (edema) and injection associated hair follicle rupture and inflammation. Hematoxylin and eosin staining of popliteal lymph nodes of rabbit G130 showed no evident inflammation or bacteria.

### Detection of treponemal DNA in skin and lymph node tissues

**Fig. 1:**
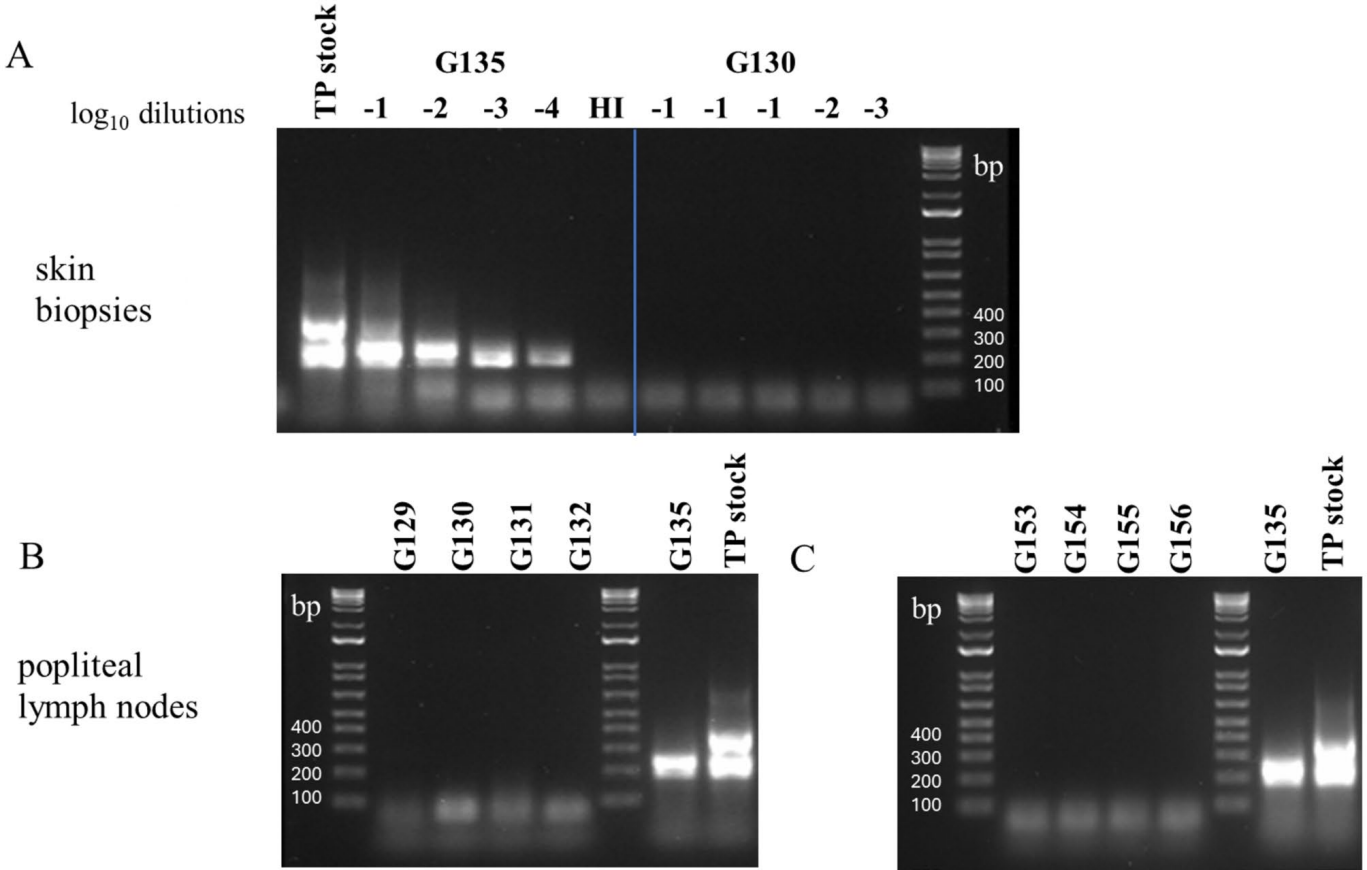
Detection of treponemal DNA in biopsy tissues. Agar gel of nested PCR products of second round of the process for skin biopsies of rabbits in Study 2 (**A**) and popliteal lymph nodes of rabbits in Study 2 (**B**) and Study 3 (**C**). (**A**) PCR products of skin from rabbit G130 inoculated with T5-Inactivated sample serially diluted from 10^–1^ to 10^–3^; G135 inoculated with T5-Control sample diluted from 10^–1^ to 10^–4^. (**B**) PCR products of lymph nodes from rabbits G129, G130, G131, G132 inoculated with T5-Inactivated sample and rabbit G135 inoculated with T5-Control sample. (**C**) PCR products of lymph nodes from rabbits G153, G154, G155, G156 inoculated with T5-Inactivated sample and rabbit G135 inoculated with T5-Control sample. HI = Heat inactivated; TP = *Treponema pallidum*.

Next, we analyzed the skin biopsies of rabbits G130 and G135 for treponemal DNA using nested PCR. The results in the Fig. 1 (panel A) show the presence of DNA isolated from skin of rabbit G135 at sites injected with T5-Control sample dilutions 10^–1^ to 10^–4^. The DNA method was very sensitive and detected the lowest dose injected estimated to have contained 50 organisms at the time of inoculation. Samples with high concentration of treponemal DNA showed a top band corresponding to the product from the first DNA amplification. Importantly, we detected no treponemal DNA at the site in rabbit G135 inoculated with heat-inactivated (HI) treponemes. This result ruled out non-specific reactivity of PCR with rabbit tissue or human platelets (the dilution vehicle). All sites injected with inactivated samples produced no treponemal DNA bands (Fig. 1, panel A, G130 lanes -1 to -3). These results strongly indicated that the indurations at the inconclusive sites were not caused by live treponemes but, likely, by an unexpected host’s reactivity or hypersensitivity to other components of the inoculum. Additionally, our results were consistent with earlier reports showing persistent DNA (at least 60 days post inoculation) at infected sites and absent at sites injected with inactivated treponemes^41^. DNA extracted from rabbit G135 lymph node showed treponemal PCR signal (Fig. 1, panel B) while no DNA was detected in lymph nodes from all rabbits, including G130, inoculated with inactivated material (G129, G130, G131, G132). Collectively, the results from histology and nucleic acid tests decisively ruled out treponemal infectivity at the three inconclusive sites and confirmed the four rabbits were syphilis-free.

In Study 3, we injected an estimated 21 million treponemes per rabbit. In previously published studies, similarly high levels of heat-inactivated bacteria inoculated intradermally into rabbits resulted in the generation of anti-treponemal antibodies, although this reactivity was delayed and weaker than that elicited by live bacteria inoculations^42,43^. Although none of the rabbits inoculated with T5-Inactivated sample seroconverted, we decided to conduct further tests to ensure no cases of syphilis infections were overlooked. We analyzed DNA from popliteal lymph nodes of rabbits inoculated with T5-Inactivated (G153, G154, G155, G156). The results confirmed no treponemal DNA in lymph nodes of serologically negative rabbits (Fig. 1, panel C). From these data, we concluded that none of the rabbits inoculated with light-inactivated treponemes had been infected.

## Discussion

Technologies to reduce or inactivate pathogens in blood have been developed and used to address residual risk of bacterial contamination of blood and are viewed as a proactive option to improve safety of blood transfusion. Bacterial contamination of platelets is particularly concerning because of room temperature storage of this blood component. We used an upcoming and promising method of pathogen inactivation that applies violet-blue 405 nm visible light to inactivate the microorganism spiked in plasma or platelets. This technology was tested extensively with multiple pathogens under experimental laboratory conditions. Studies using metabolomics analysis of platelets showed no negative impact of 405 nm light on platelet membrane integrity and platelet aggregation activity^33^. Furthermore, exposure to violet-blue light for 1 h with cumulative 360 Jcm^-2^ demonstrated four to five log_10_ reduction of several transfusion-relevant gram-positive and gram-negative bacteria spiked in human plasma^34,37^ with no significant finding in a metabolomics study^34,44,45^. These data support 405 nm light-based pathogen inactivation technology as a promising novel tool for safe and effective treatment of human ex vivo platelets and plasma.

Here we investigated the same technology against the bacterium *Treponema pallidum* spiked in human platelets at two concentrations, 10^5.7^ and 10^7.7^ treps/ml. Different from other bacteria, *Treponema pallidum* cannot be propagated in bacterial culture media and for many years this bacterium was considered unculturable. Recently, investigators have improved on earlier attempts and demonstrated that *Treponema pallidum* can be successively propagated and maintained on rabbit epithelial cell (Sf1EP) under low oxygen and other specific growth conditions^46,47^. However, the gold-standard for assaying infectivity of *Treponema pallidum* remains the propagation of the organism in an animal host, usually rabbit testis. To assess inactivation of *Treponema pallidum*, we needed to quantitate the bacteria before and after treatment. *Treponema pallidum* enumeration requires darkfield microscopy which lacks sensitivity and is unsuitable to quantify the microorganisms diluted in platelets. The conventional method to titer a *Treponema pallidum* suspension is to serially dilute the sample, inject intratesticularly each dilution into a group of rabbits and monitor rabbits for seroconversion. This method requires large numbers of animals. We titered *Treponema pallidum* samples using an alternative approach where each dilution series was injected intradermally into rabbits. This method produced an independent bacterial titration in each rabbit, reduced the number of animals needed and provided a quantitative statistical measurement of infectivity titers. One challenge of this approach was that in a few cases, such as with rabbit G130, determining the final site scores required additional investigations.

Another consideration is the sensitivity of intradermal injections and how it compares to that of the conventional testicular inoculations. We found no published studies that directly compared the two methods using the same materials. Using our data from titration of T0 samples, we estimated 50% infectious dose (ID_50_) 2500 and 1260 organisms per site in Studies 1 and 2, respectively (low titer stock) and 315 organisms per site in Study 3 (high titer stock). In all cases, our ID_50_ values were higher than 1–10 organisms/site previously reported for in vitro cultured *Treponema pallidum* organisms^46^. Human platelets could have caused this difference or our treponeme stocks, especially that with low titer, might have lost some activity after long-term storage. It is also likely that freshly prepared cultured treponemes were more infectious than our thawed organisms. We are investigating those possible scenarios. However, it should be noted that infectivity log_10_ reduction was calculated as the difference of the two titers in log_10_ and thus, it was independent from the number of treponemes in the sample. We also could not find reports of full serial dilution titrations of treponemal stocks using the intratesticular route. Here we could use data from Lin et al.^24^ showing that 100 organisms per rabbit resulted in an attach rate of 11/16 (68%) and propose that ID_50_ dose for testicular inoculations is < 100 organisms. Thus, from these limited literature data, the two inoculation modalities have sensitivities in the same order of magnitude although this conclusion should be confirmed with direct measurements.

Rabbits inoculated with light-inactivated samples either from low or high titer studies developed no syphilitic lesions and had no seroreactivity when assayed with treponemal and nontreponemal tests. These results are consistent with our in-house data for rabbits inoculated with heat-inactivated treponemes. Interestingly, bacterial DNA was not present at sites injected with as much as 5 million inactivated treponemes confirming previously reported results that dead treponemes are cleared within days^39^ possibly by macrophages [^4^, and Supplementary Fig. S3] or other mechanisms. In contrast, bacterial DNA was detected in all sites injected with T5-Control sample, even at sites with no lesions indicating that PCR was more sensitive than skin reactivity.

Violet-blue 405 nm light inactivated *Treponema pallidum* to undetectable levels by animal bioassays corresponding to > 2 log_10_ and > 4 log_10_ infectivity reduction depending on the input dose. Although treponemal load in donated blood is not known, it is likely that of the two conditions tested, the low concentration is closer to the level of contamination in the blood of a syphilis asymptomatic donor. It should be noted that our studies do not demonstrate sterility of the treated platelets since only a small portion of the total volume was assayed and a few treponemes could have survived the light-treatment and persist after 7 days storage^11^. However, unlike conventional bacteria that can grow in platelets^48^, treponemes do not propagate in platelets. Cerus Intercept Blood System technology showed reduction of spiked treponemes by > 5.9 log_10_ in plasma^25^ and ~ 7 log_10_ in platelets^24^. Our data trends are consistent with those results with two caveats: our starting titers were lower than those used in Cerus’ studies, which limited the number of log_10_ reduction that we could demonstrate and secondly, we injected 0.1 ml of sample per site compared to 1 ml inoculated into testes in Cerus’ study^24,25^. This tenfold difference in volume is in part responsible for the difference in log_10_ reduction between the studies.

In conclusion, these studies conducted using small volumes of platelets provided proof-of concept that 405 nm violet-blue light successfully inactivated *Treponema pallidum* spiked in human platelets to levels that would offer improvement in transfusion safety. Although not demonstrated in these studies, we anticipate the bactericidal effect of 405 nm violet-blue light on *Treponema pallidum* in platelets is extended also to plasma matrix, based on published data demonstrating the technology was equally effective in inactivating pathogens in both blood components^34,38,39,49^.

## Materials and methods

### Animals

All animal experiments were conducted at the Food and Drug Administration White Oak Campus, Silver Spring, Maryland with prior approval of the FDA White Oak Institutional Animal Care and Use Committee and conformed to the Guide for the Care and Use of Laboratory Animals published by the U.S. National Institutes of Health (NIH). (Animals study ASP#2022–09, LG as principal investigator.) New Zealand white rabbits (Charles River Laboratories, Saint Constant, Canada) weighing 3–4 kg with nonreactive syphilis test were used. Animals were housed individually in a room with temperature set at 16–18 °C. For intratesticular inoculations, rabbits were anesthetized with Ketamine (10 mg/kg) and Dexmedetomidine (0.025 mg/kg) injected intramuscularly followed by isoflurane (1–2%), as needed. For intradermal inoculations, rabbits were sedated with Midazolam at doses between 0.5mg/kg to 1.5 mg/kg injected intramuscularly supplemented with isoflurane, as needed. Rabbits were euthanized by intravenous or intracardiac injections of 1.5 ml of Pentobarbital solution (Euthasol, Virbac, St Louis, MO) while under deep anesthesia induced by Ketamine (50 mg/kg) and Dexmedetomidine (0.05 mg/kg) injected intramuscularly.

### Treponemal stocks

We propagated *Treponema pallidum*, Nichols strain, into naïve rabbits by intratesticular inoculations of 1 ml of treponemal stock using established protocols^50^. We monitored rabbits daily for orchitis and tested sera every 3–4 days with a treponemal test, Serodia *T. pallidum* (TP)-particle agglutination (PA) assay (Fujirebio, Malvern, PA), and a nontreponemal test, ASI rapid plasma reagin (RPR) assay (Arlington Scientific Inc., Springville, UT), according to manufacturers’ instructions. At peak orchitis, we euthanized the rabbits and collected testes aseptically. We extracted treponemes from testes in harvest medium (50% normal rabbit serum and 50% saline) for 10–15 min with shaking^50^. Next, we removed an aliquot for counting treponemes by darkfield microscopy using Nexcelom disposable slides, mixed the rest of the suspension with sterile glycerol (15–20% final concentration), aliquoted, and stored frozen as treponemal glycerol stock.

### Violet-blue 405 nm light inactivation of *Treponema pallidum*

We obtained fresh apheresis platelets collected in plasma from volunteer donors in the Research Donor Program, NIH, Bethesda, Maryland and used the day after collection. This study involving human subjects’ protocol was approved by FDA Research Involving Human Subjects Committee (RIHSC, Exemption Approval #11-036B). All methods were carried out in accordance with the relevant guidelines and regulations. Informed consent from all subjects were obtained as per National Institutes of Health, USA. We conducted three independent inactivation studies with two concentrations of *Treponema pallidum* bacteria (low and high titers) spiked in ex vivo human platelets. In the two low titer studies 1 and 2, we spiked 20 ml of human platelets with treponemal glycerol stock (1:10) for a final spike concentration of 5 × 10^5^ treponemes/ml (10^5.7^ treps/ml) and mixed gently. We removed an aliquot, performed serial dilution and intradermally inoculated the sample into rabbits within one hour of preparation (T0). We split the rest of the sample into four T-25 flasks (~ 4.5 ml/each), wrapped two flasks in aluminum foil to protect from light (negative control) and placed all four flasks in the 405 nm light treatment in a close cabinet^31^. The light source was composed of narrowband 405 nm LED arrays (FWHM∼20 nm; LED Engin, CA, USA) with appropriate thermal management and powered by LED drivers (MeanWell, Taiwan). The light source was held at 14 cm above the samples. Light exposure to 405 nm violet-blue light was for 5 h to achieve a light dose of 270 J/cm^2^ (irradiance = 15 mW/cm^2^) at room temperature and with mild agitation. Next, we pooled samples from the two exposed flasks (T5-Inactivated) and the two control flasks (T5-Control), removed an aliquot for testing, performed serial dilutions and inoculated intradermally into rabbits within one hour of preparation. In the high titer spike study, we conducted the same inactivation experiment using a treponemal stock with a titer of 5 × 10^8^ treps/ml at a final spike concentration of 5 × 10^7^ treps/ml (10^7.7^ treps/ml) (Study 3).

### Rabbit inoculations

We shaved rabbits on the back the day before inoculation, marked the sites of inoculations and kept the area shaven throughout the study. Using human platelets as the diluent, we serially diluted the three samples produced in each study (T0, T5-Inactivated and T5-Control) and immediately injected rabbits intradermally with 0.1 ml of sample per site. Each dilution was inoculated in duplicate into two adjacent sites (left and right)^46^ and typically we inoculated 5 dilutions except when otherwise noted. Each dilution series was inoculated into four rabbits resulting in a total of eight sites per dilution as indicated in the Tables in Supplementary Information. However, anticipating inactivation of the organisms by exposure to 405 nm light, instead of eight sites we inoculated T5-Inactivated at dilution 10^–1^ in 16 or 24 sites thus, increasing the probability of detecting at least one positive site. As negative controls, we inoculated platelets alone or treponemal stock heat-inactivated for 30 min at 56 °C and then diluted tenfold in platelets. For the duration of the study, we collected sera weekly and tested with Serodia TP-PA test and RPR ASI test.

We monitored rabbits and injected sites three times a week and reported the appearance of the lesions according to pre-defined scoring system. Typically, injected sites showed initial and transient erythema as a response to the intradermal injections. At sites injected with live treponemes, we observed persistent erythema that changed into induration or papule and progressed to open lesions measuring up to 20 mm and ulcers at sites inoculated with high titer samples. Changes in the appearance and size of the lesions occurred for 5–6 weeks post inoculation and we continued monitoring the rabbits for a total of 8 weeks.

### Tissue biopsies and histological evaluation

Sixty days post inoculation we euthanized rabbits inoculated with T5-Inactivated and T5-Control from Study 2. We excised approximately 80–100 mg of skin at the inoculated sites using 4 mm diameter disposable biopsy punchers with plunger (Integra LifeSciences, Mansfield, MA). We analyzed skin and popliteal lymph node tissues from rabbit G130 injected with dilutions of T5-Inactivated and G135 rabbit similarly injected with dilutions of T5-Control. We collected the tissue on the left in 10% formalin for histology and froze the matching tissue from the right site for treponemal DNA detection. We collected popliteal lymph nodes from the same rabbits for histological examination and treponemal DNA detection. Fixed tissues were cut and stained using hematoxylin and eosin (H&E) to reveal morphological changes in the skin tissue and Giemsa to examine mast cells (American Histolabs, Inc, Gaithersburg, Maryland). Selected tissues were stained with Warthin-Starry for detection of spirochetes at the injection sites (Histology Consultation Services, Inc, Everson, Washington).

### Treponemal PCR of skin tissues and popliteal lymph nodes

We performed nested PCR on DNA isolated from skin and lymph nodes of G130 and control animals from Study 2. Similarly, we excised popliteal lymph nodes from rabbits injected with T5-Inactivated in Study 3 and processed the tissues for treponemal DNA detection. We isolated total DNA from tissues using Quick-DNA Midiprep Plus Kit (Zymo Research, Irvine, CA) according to the vendor’s instructions. Nested PCR targeting the polA gene was performed initially with inner PolA1 (sense: 5′ -TGCGCGTGTGCGAATGGTGTGGTC-3′ and anti-sense: 5′ -CACAGTGCTCAAAAACGCCTGCACG-3′) in a total volume of 50 μl. Each reaction consisted of 10 μl of DNA, 40 μl of PCR master mix 36 µl AmpliTaq Gold 360 Master Mix (Thermo Fisher Scientific, Waltham, MA) and 2 µl of each primer. In the second round of PCR, 5 μl of the first reaction product was used in a total volume of 50 μl of reaction mixture with PolA2 the sense primer 5′-GGATTGCATCCGCACGATAC-3′ and the anti-sense primer 5′-CAGCAGATGCAGATACCCCA-3′^51^. DNA isolated from 100 μl of *Treponema pallidum* glycerol stock using QIAamp DNA Mini Kit (Qiagen, Hilden, Germany) served as positive control for nested PCR.

### Statistical analysis

We scored each site as reactive (positive) or nonreactive (negative), we combined the results for all rabbits inoculated with the same sample (usually four rabbits) and based on the ratio reactive/nonreactive sites as a function of sample dilution, we calculated the ID_50_ per volume inoculated (0.1 ml) using the Reed-Muench method^52^, adjusted the titer to 1 ml of inoculum and reported it as ID_50_/ml. In the cases of T5-Inactivated for which all sites were negative, we assumed the worse-case where tenfold higher concentration would have resulted in all sites being reactive. Log_10_ reduction was calculated as the difference in log_10_ titers of the untreated (T5-Inactivated) and treated (T5-Control) samples.

## Electronic supplementary material

Below is the link to the electronic supplementary material.

Supplementary Material 1

## Data Availability

All data generated during this study are available upon request to the corresponding author. We report here the data that support the findings of this study and its Supplementary Information files.
