# Supplementary material for: 405 nm light microbicidal efficacy on Treponema pallidum spiked in ex vivo human platelets
Source: Sci Rep. 2025 Jun 6;15:19893. doi: 10.1038/s41598-025-03230-1 (PMC12144293; doi:10.1038/s41598-025-03230-1)
Supplement: Supplementary file 1 — Supplementary Material 1 [file 41598_2025_3230_MOESM1_ESM.docx]

**Supplementary information**

**405 nm light microbicidal efficacy on *Treponema pallidum* spiked in *ex vivo* human platelets**

Oksana Yakovleva, Teresa Pilant, Pravin Kaldhone, Joseph Jackson, David Rotstein, Caitlin Stewart, John Anderson, Scott MacGregor, Michelle Maclean, Luisa Gregori, Chintamani Atreya.

Included:

Supplementary Table S1

Supplementary Table S2

Supplementary Table S3

Supplementary Figure S1

Supplementary Figure S2

Supplementary Figure S3

Supplementary Figure S4

Table S1. Summary scoring of inoculated sites in Study 1. In Study 1, we spiked with low titer of treponemes and we injected rabbits with serial dilutions of T0, T5-Inactivated and T5-Control samples. The table represents scoring 38 days post inoculation.

|  |  | T0 | | | | T5-Inactivated | | | | | T5-Control | | | |
| --- | --- | --- | --- | --- | --- | --- | --- | --- | --- | --- | --- | --- | --- | --- |
| Trep/site | Logs dilut^*^ | G113 | G114 | G115 | G116 | | G117 | G118 | G120 | G121 | | G122 | G123 | G124 |
| Sites |  | L R | L R | L R | L R | | L R | L R | L R | L R | | L R | L R | L R |
| 50,000 | -1 | **P P** | **P P** | **P P** | **P P** | | N N  N N | N N  N N | N N  N N | N N  N N | | **P P** | **P P** | **P P** |
| 5,000 | -2 | **P P** | **P** N | **P** N | **P P** | | N N | N N | N N | N N | | **P P** | **P** N | **P** N |
| 500 | -3 | N N | N N | N N | N N | | N N | N N | N N | N N | | N N | N N | N N |
| 50 | -4 | N N | N N | N N | N N | | - | - | - | - | | N N | N N | N N |
| 5 | -5 | N N | N N | N N | N N | | - | - | - | - | | N N | N N | N N |
| Heat inact |  | - | - | - | - | | N N | N N | N N | N N | | - | - | - |


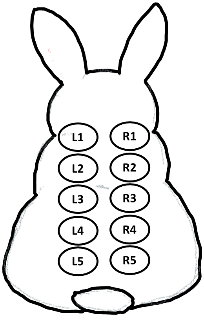


^*^ All dilutions refer to treponemal stock. log_10_ dilution -1 represents 10-fold dilution of stock into human platelets which is the same as undiluted spiked platelets.

L = left side; R = right side

P = positive; N = negative

Table S2. Summary scoring of inoculated sites in Study 2. In Study 2, we spiked with low titer of treponemes and we injected rabbits with serial dilutions of T0, T5-Inactivated and T5-Control samples. The table represents scoring 38 days post inoculation.

|  |  | T0 | | | | T5-Inactivated | | | | T5-Control | | | |
| --- | --- | --- | --- | --- | --- | --- | --- | --- | --- | --- | --- | --- | --- |
| Trep/site | log_10_ dilut^*^ | G125 | G126 | G127 | G128 | G129 | G130 | G131 | G132 | G133 | G134 | G135 | G136 |
| Sites |  | L R | L R | L R | L R | L R | L R | L R | L R | L R | L R | L R | L R |
| 50,000 | -1 | **P P** | **P P** | **P P** | **P P** | N N  N N  N N | N N  N N  N N | N N  N N  N N | N N  N N  N N | **P P** | **P P** | **P P** | **P P** |
| 5,000 | -2 | **P P** | **P P** | **P** N | **P P** | N N | N N | N N | N N | **P** N | **P P** | **P P** | **P P** |
| 500 | -3 | **P P** | N N | N N | N N | N N | N N | N N | N N | N N | N N | N N | N N |
| 50 | -4 | N N | N N | N N | N N | - | - | - | - | N N | N N | N N | N N |
| 5 | -5 | N N | N N | N N | N N | - | - | - | - | N N | N N | - | - |
| Heat inact |  | - | - | - | - | - | - | - | - | - | - | N N | N N |


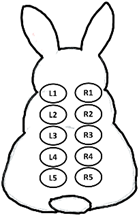


^*^ All dilutions refer to treponemal stock. log_10_ dilution -1 represents 10-fold dilution of stock into human platelets which is the same as undiluted spiked platelets.

N = Sites with initial indeterminate results that successively were resolved as negative (N).

L = left side; R = right side

P = positive; N = negative

Table 3. Summary scoring of inoculated sites in Study 3. In Study 3, we spiked with high titer of treponemes and we injected rabbits with serial dilutions of T0, T5-Inactivated and T5-Control samples. The table represents scoring 44 days post inoculation.

|  |  | titration | | | | T5-Inactivated | | | | T5-Control | | | |
| --- | --- | --- | --- | --- | --- | --- | --- | --- | --- | --- | --- | --- | --- |
|  |  | G149 | G150 | G151 | G152 | G153 | G154 | G155 | G156 | G157 | G158 | G159 | G160 |
| Trep/site | Logs  Dilut^*^ | L R | L R | L R | L R | L R | L R | L R | L R | L R | L R | L R | L R |
| 5,000,000 | -1 | - | - |  |  | N N  N N | N N  N N | N N  N N | N N  N N | - | - | - | - |
| 500,000 | -2 |  |  |  |  | N N | N N | N N | N N | - | - | - | - |
| 50,000 | -3 | **P P** | **P P** | **P P** | **P P** | N N | N N | N N | N N | **P P** | **P P** | **P P** | **P P** |
| 5,000 | -4 | **P P** | **P P** | **P P** | **P P** | N N | N N | N N | N N | **P P** | N  **P** | **P P** | **P** N |
| 500 | -5 | **P** N | N **P** | **P** N | **P P** | - | - | - | - | N N | N **P** | N **P** | N N |
| 50 | -6 | N N | N N | N N | N N | - | - | - | - | N N | N N | N N | N N |
| 5 | -7 | N N | N N | N N | N N | - | - | - | - | N N | N N | - | - |
| Platelets | - | - | - | - | - | - | - | - | - | - | - | N N | N N |


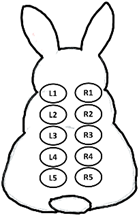


^*^ All dilutions refer to treponemal stock as dilution 0. log_10_ dilution -1 represents 10-fold dilution of stock into human platelets which is the same as undiluted spiked platelets.

L = left side; R = right side

P = positive; N = negative


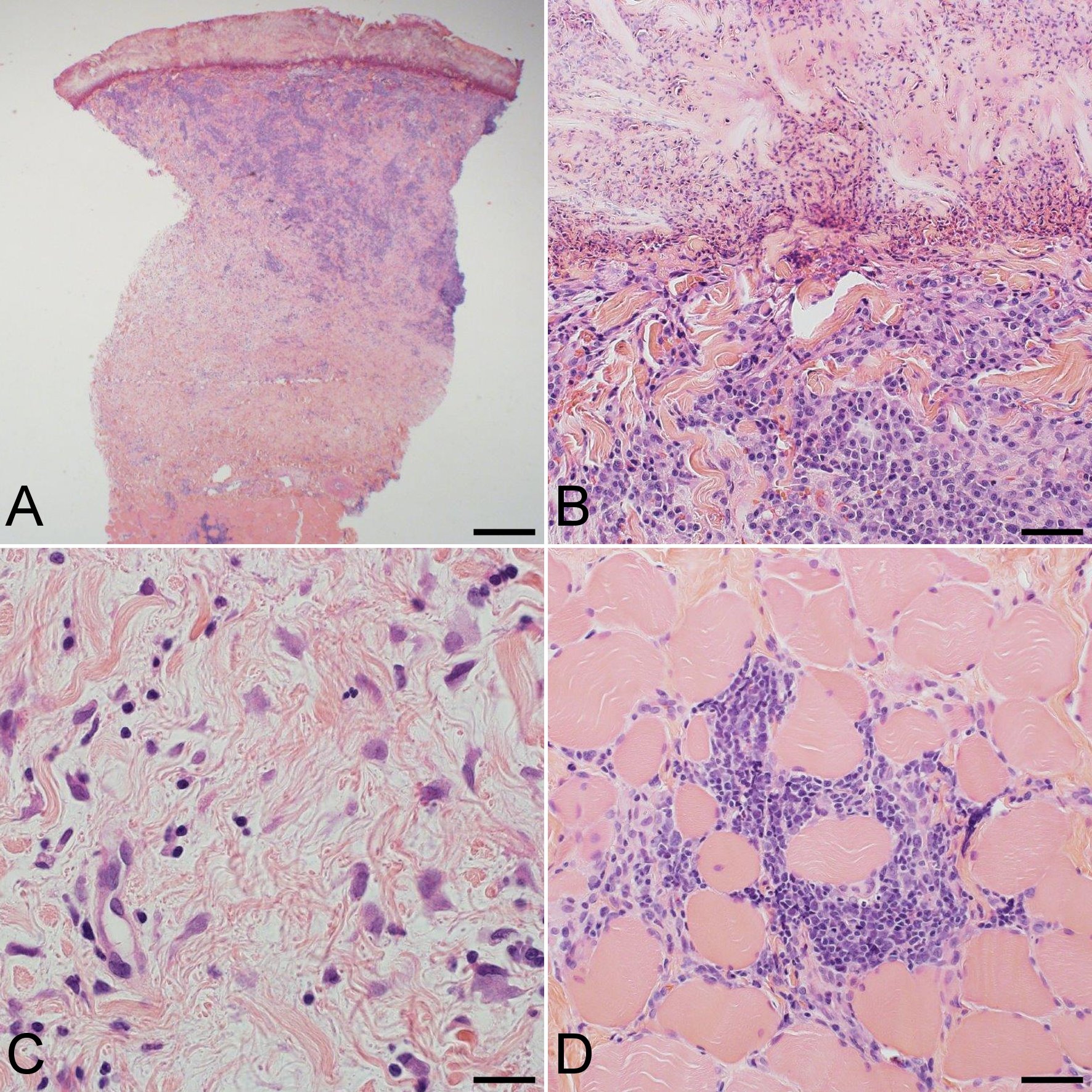


Figure S1. Hematoxylin and eosin staining of rabbit G135 inoculated with T5-Control dilution 10^-1^. (A) Epithelial ulceration and the dermis are infiltrated by inflammatory cells. Magnification 2X, bar = 500 µm (B) The epithelium is covered by a thick crust of necrotic debris with infiltrating heterophils and the dermis is expanded by mononuclear cells including lymphocytes, plasma cells, and macro-phages. Magnification 20X, bar = 50 µm. (C) Dermal collagen bundles are separated by clear spaces (edema). Magnification 40X, bar = 20 µm. (D) There is a focal infiltrate of lymphocytes, plasma cells, and macrophages within the skeletal muscle. Magnification 20X, bar = 50 µm.


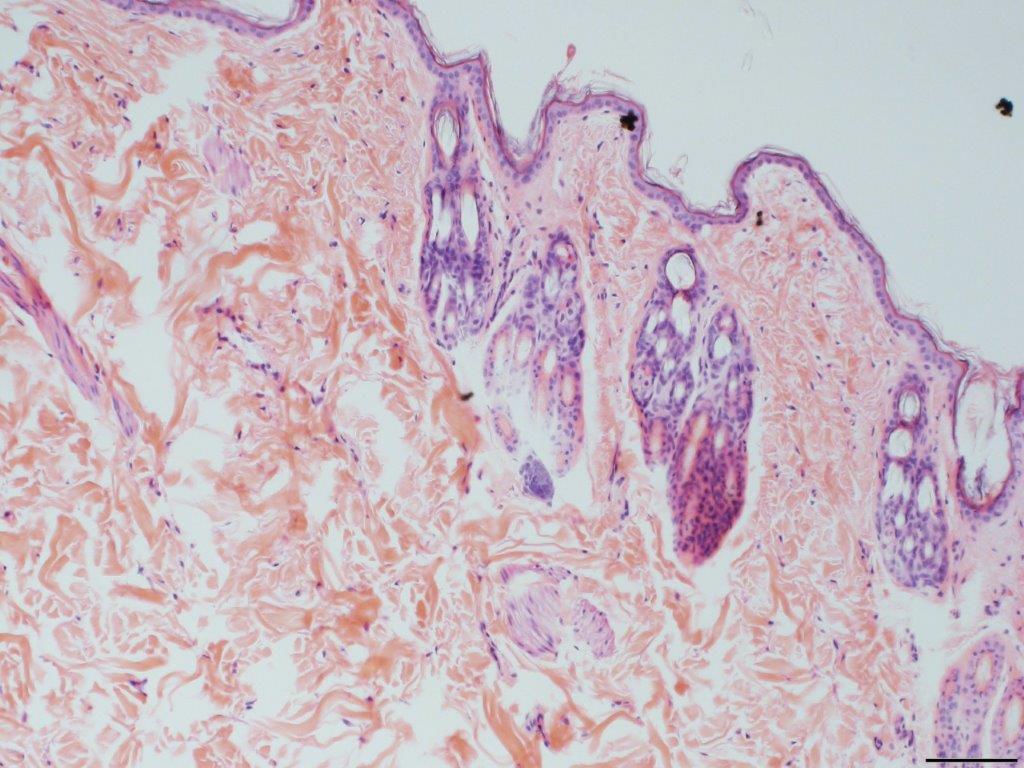


Figure S2. Hematoxylin and eosin staining of rabbit G130 inoculated with T5-Inactivated dilution 10^-3^. There is no inflammation, ulceration, hemorrhage, or necrosis. Magnification 10X, bar = 100 µm.


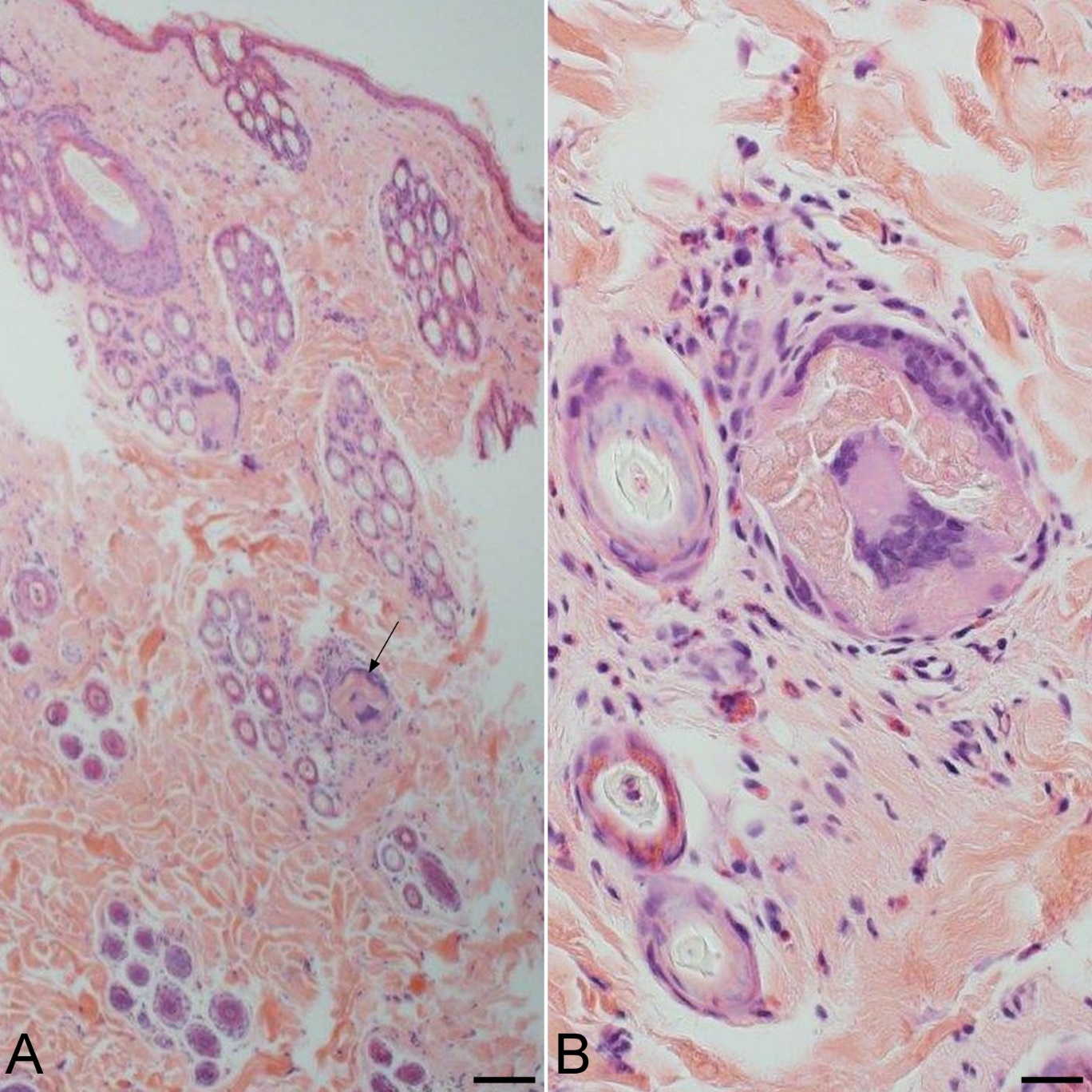
 Figure S3. Hematoxylin and eosin-stated section of skin from rabbit G130 inoculated with T5-Inactivated dilution 10^-1^. (A) The epithelium is intact. There is minimal edema and inflammation. There is a single ruptured follicle (arrow). Magnification 4X, bar = 200 µm. (B) The ruptured follicles have fragmented hair, macrophages, and a multinucleated giant cell (macrophage). Magnification 20X, bar = 50 µm.


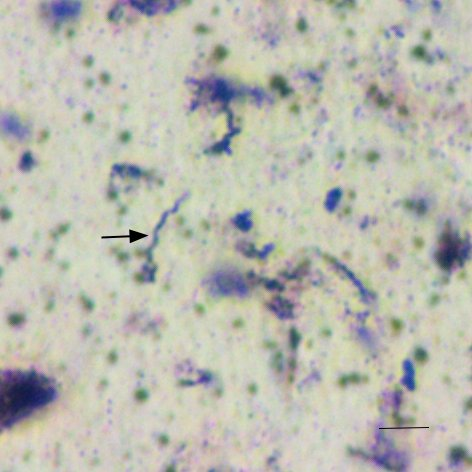


Figure S4. Warthin-Starry stained section of skin. Skin from G135 inoculated with untreated T5-Control sample at 10^-1^ dilution. The staining shows treponemes in blue (arrow). The scale bar = 10 µm.
